# Factors Associated with the Breast Cancer Diagnostic Interval across Five Canadian Provinces: A CanIMPACT Retrospective Cohort Study

**DOI:** 10.3390/cancers15020404

**Published:** 2023-01-07

**Authors:** Arlinda Ruco, Patti A. Groome, Mary L. McBride, Kathleen M. Decker, Eva Grunfeld, Li Jiang, Cynthia Kendell, Aisha Lofters, Robin Urquhart, Khanh Vu, Marcy Winget

**Affiliations:** 1 Interdisciplinary Health Program, St. Francis Xavier University, Antigonish, NS B2G 2W5, Canada; 2 Women’s College Hospital, Toronto, ON M5S 1B2, Canada; 3 Cancer Care and Epidemiology, Queen’s Cancer Research Institute, Kingston, ON K7L 3N6, Canada; 4 British Columbia Cancer Agency, Vancouver, BC V5Z 4E6, Canada; 5 CancerCare Manitoba Research Institute, Winnipeg, MB R3E 0V9, Canada; 6 Department of Community Health Sciences, University of Manitoba, Winnipeg, MB R3T 2N2, Canada; 7 Department of Family and Community Medicine, University of Toronto, Toronto, ON M5S 1A1, Canada; 8 Ontario Institute for Cancer Research, Toronto, ON M5G 0A3, Canada; 9 Ontario Medical Association, Toronto, ON M5S 3C1, Canada; 10 Department of Surgery, Dalhousie University and Nova Scotia Health Authority, Halifax, NS B3H 4R2, Canada; 11 Department of Community Health and Epidemiology, Dalhousie University, Halifax, NS B3H 4R2, Canada; 12 School of Public Health, University of Alberta, Edmonton, AB T6G 2R3, Canada; 13 Stanford School of Medicine, Stanford, CA 94305, USA

**Keywords:** diagnostic interval, breast cancer

## Abstract

**Simple Summary:**

The breast cancer diagnostic process is a stressful period for patients. We looked at the length of the diagnostic interval within and across five Canadian provinces: British Columbia, Alberta, Manitoba, Ontario, and Nova Scotia. Our analysis was conducted separately for those who had their cancer detected through the respective provincial screening program versus those outside of the provincial screening program (symptom-detected). The diagnostic interval was shorter for patients who had their cancer detected through the screening program. Interprovincial diagnostic interval variation was 17 and 16 days for screen- and symptom-detected patients, respectively, at the median, and 14 and 41 days, respectively, at the 90th percentile. The diagnostic interval was longer for those with more comorbid disease among the symptom-detected group. Screen-detected patients living in rural areas also had a longer diagnostic interval. Having a regular primary care provider was not associated with a shorter diagnostic interval.

**Abstract:**

The cancer diagnostic process can be protracted, and it is a time of great anxiety for patients. The objective of this study was to examine inter- and intra-provincial variation in diagnostic intervals and explore factors related to the variation. This was a multi-province retrospective cohort study using linked administrative health databases. All females with a diagnosis of histologically confirmed invasive breast cancer in British Columbia (2007–2010), Manitoba (2007–2011), Ontario (2007–2010), Nova Scotia (2007–2012), and Alberta (2004–2010) were included. The start of the diagnostic interval was determined using algorithms specific to whether the patient’s cancer was detected through screening. We used multivariable quantile regression analyses to assess the association between demographic, clinical and healthcare utilization factors with the diagnostic interval outcome. We found significant inter- and intra-provincial variation in the breast cancer diagnostic interval and by screen-detection status; patients who presented symptomatically had longer intervals than screen-detected patients. Interprovincial diagnostic interval variation was 17 and 16 days for screen- and symptom-detected patients, respectively, at the median, and 14 and 41 days, respectively, at the 90th percentile. There was an association of longer diagnostic intervals with increasing comorbid disease in all provinces in non-screen-detected patients but not screen-detected. Longer intervals were observed across most provinces in screen-detected patients living in rural areas. Having a regular primary care provider was not associated with a shorter diagnostic interval. Our results highlight important findings regarding the length of the breast cancer diagnostic interval, its variation within and across provinces, and its association with comorbid disease and rurality. We conclude that diagnostic processes can be context specific, and more attention should be paid to developing tailored processes so that equitable access to a timely diagnosis can be achieved.

## 1. Introduction

The cancer diagnostic process is a time of great anxiety for patients [1]. If the time from the first cancer-related encounter with the health care system to diagnosis (diagnostic interval) is long, patient anxiety can increase and ultimately, the probability of cure could be compromised [1,2,3]. The time consuming aspects of the diagnostic process include waiting for tests and consultations, test duplication, and a general lack of care coordination given the variety of health professionals involved [4]. These factors can also be affected by other strains to the health care system, including most recently the COVID-19 pandemic [5,6]. A systematic review of the association between time to diagnosis and outcomes for those with symptomatic cancers found some evidence suggesting that longer intervals are associated with poorer outcomes, especially mortality [3].

Current Canadian breast cancer guidelines recommend that at least 90% of patients receive a definite diagnosis within five weeks of their abnormal screen result if no tissue biopsy is performed or within seven weeks if tissue biopsy is performed [7]. While all citizens are promised equitable access to high quality care via the Canada Health Act with care delivery through provincial/territorial-level health care systems [8], some delays are still prevalent [9,10,11]. 

Patients present with breast cancer either through screening or symptomatically. All Canadian provinces and most territories have organized breast cancer screening programs that identify asymptomatic individuals who are eligible for screening, manage screening invitations and coordinate diagnostic follow-up and recall. All eligible individuals are able to access screening in this way. Opportunistic screening, which is when a healthcare provider orders a mammogram for an asymptomatic patient, can also occur outside of some provinces’ organized cancer screening programs. Alternatively, some patients present to their physician when they have signs and/or symptoms of disease. The factors associated with the length of the diagnostic interval can vary for screen- and non-screen detected cancers [11]. Thus, the study of these two presentations should occur separately. 

As part of a broader study of the integration between primary care and oncology services relevant to breast cancer [12,13,14,15], we undertook a multi-province study to examine the care of breast cancer patients during the diagnostic interval. The specific objectives of this study were to quantify inter- and intra-provincial variation in diagnostic intervals and explore factors related to the variation. 

## 2. Materials and Methods

This was a multi-province retrospective cohort study using linked cancer registry, clinical and administrative health data. The work outlined in this manuscript was carried out in parallel across five Canadian provinces: British Columbia, Alberta, Manitoba, Ontario, and Nova Scotia. Approval was obtained from all relevant institutional research ethics boards, as well as all relevant data access and privacy review bodies.

### 2.1. Study Population 

All provinces created de novo datasets for the purpose of this study, except for Alberta, which used an existing cohort. We included all females with a diagnosis of histologically confirmed invasive (behavior = 3, ICD-O) breast cancer (174.0 to 174.9 ICD-9) for British Columbia (2007–2010), Manitoba (2007–2011), Ontario (2007–2010), Nova Scotia (2007–2012), and Alberta (2004–2010) [15]. For patients with more than one cancer diagnosis on the same day, we used a hierarchy to pick which record to keep: the case with the highest stage; or highest histology priority; or first malignancy number. We excluded patients who did not have a valid unique linkage identifier; were not a resident of the province; were diagnosed with in situ or stage 0 breast cancer; had in situ breast cancer history or any cancer history (except for non-melanoma skin cancer); and those whose histology met a list of exclusions that constituted rare presentations such as Paget disease of breast, myeloid sarcoma, or atypical meningioma. We also excluded those who did not have provincial health insurance coverage for at least 6 months pre-diagnosis as this look-back window was required to ascertain the diagnostic interval.

### 2.2. Diagnostic Interval

The diagnostic interval was defined as the time from the order date of the first breast cancer-related investigation or the screening date to the date of breast cancer diagnosis [12]. This definition excludes the primary care interval, as defined in the Aarhus statement [16], which we were unable to operationalize across five provinces [12]. As such, our diagnostic interval in the non-screened group are underestimates for the small number of patients who presented with atypical symptoms [16]. In all provinces, the diagnosis date was obtained from the provincial, population-based cancer registry [15]. 

To identify the start of the diagnostic interval, we first determined whether the patient’s cancer was screen-detected (i.e., asymptomatic) or non-screen-detected (i.e., symptomatic) based on breast-related encounters within 6 months prior to the diagnosis date. This lookback window was selected based on previous validation and research studies [9,12,17]. Patients were defined as screen-detected if they had abnormal mammograms within the provincial breast cancer screening program or opportunistic mammograms within 6 months prior to the diagnosis date [15]. Opportunistic mammographs include those occurring outside of the formal organized cancer screening program, usually because the provider wants screening to occur at an earlier age or different frequency than recommended by the provincial screening program or because the patient does not have access to the program. A specific billing code is used in these cases to indicate that the procedure is for screening, not symptoms. The date of abnormal screen was used as the start of the diagnostic interval. All patients without an abnormal screen were defined as non-screen-detected. For these patients, physician billing claims and the Canadian Institute of Health Information’s (CIHI) Discharge Abstract Database (DAD) (i.e., hospitalization data) were used to identify relevant diagnostic tests, health care encounters and their dates. We defined the start of the diagnostic interval for non-screen-detected patients as the first investigation order date within 6 months prior to (including) the date of diagnosis, consistent with prior research [9,12,17]. Specifically, we used: (a) the order date of the first diagnostic test (Nova Scotia); (b) the last visit (up to 6 months prior to the first diagnostic test) to the referring doctor who ordered the first diagnostic test. If the referring doctor information was missing, the test date was used (British Columbia and Ontario); or (c) the last visit (up to 6 months prior to the first diagnostic test) to a primary care physician before the first test. If the primary care visit was missing, the test date was used (Alberta) [12]. In Manitoba, all conditions were considered, and the earliest date used. In Ontario and Nova Scotia, for the very small subset of patients with no tests, we took the earliest date of breast-related encounters (as indicated by diagnosis codes) captured in physician billing claims and the CIHI-DAD database in 6 months before (and including) the date of diagnosis. The relevant diagnosis codes are listed in Appendix A.

### 2.3. Study Variables

Age at diagnosis was calculated based on date of birth captured in the cancer registries and in provincial health insurance client registries. Comorbidity was assessed using the Johns Hopkins ACG System [18,19], which assigned patients to Aggregated Diagnosis groups (ADGs) based on diagnosis codes within physician billing claims and CIHI-DAD data from the 6 to 30 months prior to the breast cancer diagnosis. We created categories of the total Aggregate Diagnosis Group (ADG) count based on frequency distribution. In Alberta, the Charlson comorbidity index was used instead of ADGs to account for patient comorbidity as Alberta did not have access to the John Hopkins ACG System. 

Area-level income and rurality from the 2006 Census were assigned using the Postal Code Conversion File [20] and patient’s postal code at diagnosis. Income quintile cuts were provincially based. Because of the inter-relationship between SES and rurality, we created a combined variable with six categories: low SES rural (quintile 1), medium SES rural (quintiles 2,3,4), high SES rural (quintile 5), low SES urban (quintile 1), medium SES urban (quintiles 2,3,4), and high SES urban (quintile 5). Continuity of primary care was assessed using the Usual Provider of Care Index (UPC) [21], which uses primary care physician billing claims in the 6 to 30 months prior to the cancer diagnosis date to determine the proportion of visits to the most-often-visited primary care physician. This index was calculated only for patients with at least 3 visits, with perfect continuity defined as a score of 1 and high continuity defined as >0.75. Assignment into the provincial healthcare regions was based on patient diagnosis code at diagnosis. Further details on the derivation of these variables are reported elsewhere [12]. 

### 2.4. Analysis

Parallel datasets were created in each province and analyzed separately using common dataset creation and analysis plans [12]. Province-specific analyses are presented stratified by detection method (screened, non-screened). Inter-provincial statistical comparisons were not possible because data could not travel out of province and therefore could not be combined into one dataset. We calculated descriptive statistics to describe the study cohort across provinces and present the diagnostic interval for screen- and non-screen- detected cancers at the 25th, 50th, 75th, and 90th percentile. We also calculated the inter- and intra-provincial (across healthcare regions) variation in the diagnostic interval median and 90th percentile stratified by detection method. We used multivariable quantile regression analyses at the median and 90th percentile to assess the association between demographic, clinical and healthcare utilization factors with the outcome of diagnostic interval in days. Missing data represented a very small proportion (<0.3%) and were removed from multivariable analysis as this was unlikely to skew the results. We report *p*-values < 0.001 to account for the number of within province multiple comparisons. All statistical analyses were performed using statistical software SAS 9.4 (SAS Institute, Cary, NC, USA). 

## 3. Results

Table 1 presents the population characteristics by province in the screen-detected group. Briefly, 24,281 women had a screen-detected breast cancer across the five provinces with the majority aged 50-69 years. Comorbidity burden was highest in Nova Scotia and lowest in British Columbia. Living in a medium SES urban area was the most common SES-rurality category in each province, ranging from 40% in Nova Scotia to 54% in British Columbia. Continuity of care was highest in Nova Scotia with 62% of the cohort having a UPC > 0.75 compared to a low of 44% in Alberta. 

Table 2 presents the population characteristics by province in the non-screen-detected group. A total of 53,025 women had a non-screen-detected breast cancer across the five provinces. Except for the <40 group, age was somewhat evenly distributed across the age categories in all provinces. Consistent with the screen-detected group, 40–53% were classified as living in a medium SES urban neighborhood and comorbidity burden was lowest in British Columbia. Continuity of care was highest in Ontario and Nova Scotia with 54% having a UPC > 0.75 and lowest in Alberta at 38%.

**Figure 1:**
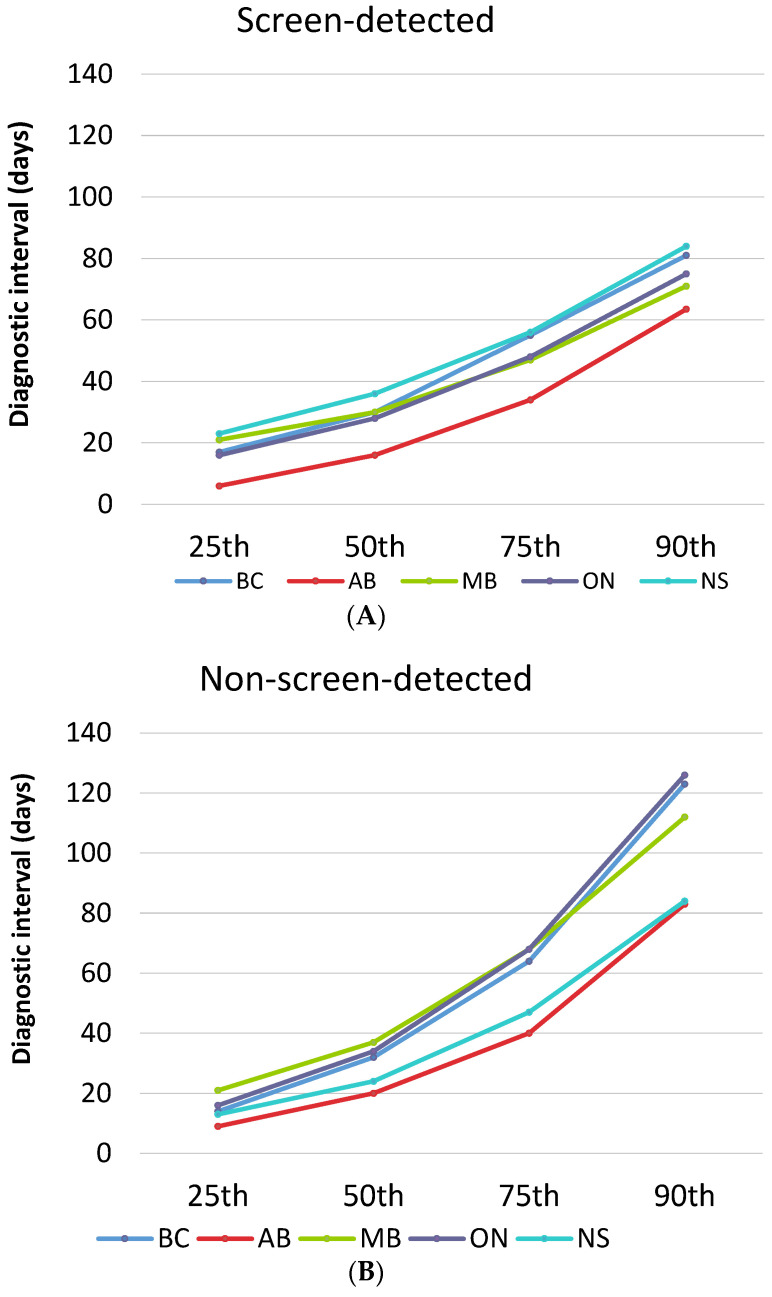
Diagnostic interval across provinces stratified by detection method. (**A**) Screen-detected cancers; AB = Alberta; BC = British Columbia; MB = Manitoba; NS = Nova Scotia; ON = Ontario. (**B**) Non-screen-detected cancers.

Figure 1 plots the diagnostic interval cumulative distribution with values at the 25th, 50th, 75th and 90th percentiles by province and stratified by detection method. Looking across all provinces, screen-detected patients waited a median of 19 to 36 days for a diagnosis while non-screen-detected patients waited a median of 21 to 37 days (Table 3). Those with a non-screened-detected cancer had a longer median diagnostic interval than those with a screen-detected cancer in all provinces except for Nova Scotia, where the median was higher for screen-detected cancers (36 versus 24 days, respectively). The diagnostic interval 90th percentile was considerably longer for those with a symptomatic cancer except for Nova Scotia where the value was almost identical in the screened (84 days) and non-screen-detected groups (85 days). In addition to interprovincial variation, there was also variation within each province (Appendix B and Appendix C). For screen-detected cancers, the highest intra-provincial variation was found in Alberta with a 34-day difference in the median diagnostic interval (Appendix D). For non-screen-detected cancers, the highest intra-provincial variation was found in Manitoba with a difference of 26 days in the median diagnostic interval (Appendix D). The lowest interprovincial variation in the median diagnostic interval for non-screen-detected cancers was found in Ontario with a difference of 15 days (Appendix D). 

Table 4 and Table 5 present the results of the multivariable quantile regression model for those with a screen-detected and non-screen-detected cancer. There was no evidence of longer diagnostic intervals in older adults (75+) among those with a screen-detected cancer. Younger (<50 years of age) screen-detected patients in Ontario had a significantly shorter diagnostic interval compared to those aged 60–69. For those with a non-screen detected cancer in Ontario, older adults (75+) had a significantly shorter median diagnostic interval by six days. No significant differences were found in the other provinces. 

There was an association between increasing comorbid disease status and longer median diagnostic intervals in the non-screen-detected group across the four provinces with data available. This pattern was not observed in the screened group.

Where significant differences existed, screen-detected patients in rural settings typically had longer diagnostic intervals than those in urban settings. The largest median difference between urban and rural regions was in Nova Scotia. The median time to diagnosis was 19 days longer for those living in low SES rural areas compared to those in high SES, urban areas. This pattern was not observed in the non-screen-detected group.

Patients with high continuity of primary care had similar median diagnostic intervals to patients with lower continuity of care in both the screened and non-screened groups in most provinces.

## 4. Discussion

We found clinically meaningful inter and intra-provincial variation in the breast cancer diagnostic interval and by detection method; patients who presented symptomatically had longer intervals than screen-detected patients. Interprovincial diagnostic interval variation was 17 and 16 days for screen- and non-screen-detected patients, respectively, at the median, and 14 and 41 days, respectively, at the 90th percentile. Intra-provincial jurisdictional variation was wider reaching a maximum of 34 days at the median and 127 days at the 90th percentile in the screen-detected group in Alberta. We saw no evidence of longer diagnostic delay in older adults. There was an association of longer diagnostic intervals with increasing comorbid disease in all provinces in symptomatic-detected patients but not screen-detected. Longer intervals were observed across most provinces in screen-detected patients living in rural areas. Having a regular primary care provider was not associated with a shorter diagnostic interval.

Our data show that more than 25% of breast cancer patients are not being diagnosed within the current Canadian benchmark of seven weeks [7]. Our findings are consistent with prior work. For example, in a study exploring the length of the diagnostic interval among 3920 patients who underwent a screening or diagnostic mammography, the range was 1–89 days without tissue sampling and 1–128 days for those requiring tissue sampling [22]. The 90th percentile, however, was observed to be lower than in our study at 63 days [22]. Neither our study nor a scoping review identifying predictors of delayed diagnoses in symptomatic breast cancer found a relationship between patient age and length of diagnostic interval [11,23]. However, data from a recent scoping review looking at factors associated with longer diagnostic interval in low- and middle-income countries found that low health literacy, presence of comorbidities, unemployment, lower SES, older age, being unmarried, and residing far from a health facility or having a longer travel time were associated with a longer diagnostic interval [24].

Residents in rural areas were more likely to experience a longer diagnostic interval. This finding was observed among screen-detected patients in Nova Scotia, British Columbia, Ontario, and Alberta with a difference of between five and 19 days in the median between those in low SES rural areas and high SES urban areas. This finding may be partially explained by health system delivery processes and limited access to specialized care in rural areas for screening and diagnostic services or to the longer travel time if residing further from a health facility as suggested by prior work [24]. However, symptom-detected rural residents in Manitoba had shorter diagnostic intervals than urban residents. A further examination of the context and screening guidelines and policies present in Manitoba may provide useful information for other regions to improve timely diagnosis in rural areas. Prior work, not from Manitoba, suggested that those living in rural areas may actually receive more rapid diagnostic assessments to minimize the impact on patients having to travel longer distances to access care while those living in urban areas would be less likely to receive rapid assessments as they live in close proximity to care facilities [25]. We were not able to combine datasets across provinces to calculate the average and look at the impact of factors such as age and continuity on overall intervals. However, we think that it is of more interest to examine the similarities and differences in these associations across provinces as health care quality and access are context specific.

We found that those with a non-screen-detected cancer have a longer median diagnostic interval than those with a screen-detected cancer in most provinces (all except NS) in alignment with prior work [25]. This difference will partly be due to a later diagnostic interval start date for the screened group, that being an abnormal mammogram. Women with screen-detected cancers may also be more likely to undergo organized assessment through an organized (versus opportunistic) screening program where referrals for diagnostic follow-up after an abnormal mammogram are centrally coordinated [26,27]. Prior work from Ontario has shown that women diagnosed at an organized assessment center through the provincial breast screening program were almost twice as likely to receive their diagnosis within seven weeks compared to those who underwent usual care with a difference of 11 days in the median diagnostic interval between the two groups [27]. Similar findings were also reported by Jiang et al. [9] who explored the difference in the diagnostic interval between patients diagnosed through a specialized assessment unit in comparison to usual care. Crivellaro et al. [22] also found that diagnostic care provided via a rapid diagnostic unit during the COVID-19 pandemic was faster than diagnostic care before the pandemic outside of a rapid assessment center. In contrast to all other provinces, the median diagnostic interval in NS was greater for the screen detected group than the non-screen detected group. This can be attributed to breast imaging in the province, whereby breast screening and diagnostic imaging are centrally coordinated and diagnostic workups are prioritized when demands for imaging are high.

We found variation both within and across provinces with regards to the diagnostic interval of both screen-detected and non-screen detected breast cancers. This finding is also consistent with prior work including regional variation reported by Plotogea et al. [28] and variation across provinces with regards to the diagnostic interval [29,30]. We also found that a consistent relationship with a primary care provider does not translate into a shorter diagnostic interval for either screen-detected or non-screen detected breast cancer. These findings may be explained by diagnostic delays being influenced by system-level factors and capacity, as well as by organization of services, rather than by primary care providers. For example, in NS, these findings likely reflect the role of the Nova Scotia Breast Screening Program in coordinating follow-up imaging and investigations for breast abnormalities identified via screening or diagnostic imaging (i.e., patients do not need to be referred back to a primary care physician to arrange subsequent investigations). Though outside the scope of this manuscript, it would also be important to explore the association between diagnostic delay and outcomes. Some evidence suggests that there is an association between time to diagnosis and outcomes, such as higher mortality for symptomatic breast cancer [3].

There are several strengths and limitations to this study. We used population-based cohorts and developed common analytic approaches to help ensure consistency and comparability across provinces. Team members in each province had extensive experience working with registry and administrative health data and were familiar with the nuances of local data holdings. Limitations of this work include using administrative health data that are routinely collected for administrative and managerial purposes rather than for research purposes. As such, we were limited to the variables captured in these databases, which are also prone to some coding errors and/or missing data. However, the proportion of missing data in our study was very small and using these data sources allow for population-level evidence. While there were some differences in the structure of the provincial registries and databases that we used, we made every effort to ensure that our study variables were similarly defined, and our that our diagnostic interval algorithms were equivalent. These methods were informed by our prior work [12,17]. Our diagnostic interval definition does not fully adhere to the Aarhus definition [16] as we used the first test-ordering as our start date rather than first clinical presentation. This would have biased the diagnostic interval downward for the small subset of patients whose health care provider did not order a diagnostic test at that time [31]. We used an arbitrary six-month lookback period for the diagnostic interval determination. This choice was based on previous work in Ontario that shows that more than 90% of cases were covered using this timeframe. This choice would also have biased our diagnostic intervals downward in the small number in the symptomatic group whose diagnostic interval was beyond six months. The use of cancer registry data for cohort identification meant we were unable to study the diagnostic interval of patients being investigated for breast cancer who ended up not having breast cancer. As such, the attribution of our findings is restricted to persons who are ultimately diagnosed with breast cancer. This includes those whose first test may have been a false negative if it occurred within six months. The diagnostic interval is known to vary by cancer stage [4,28,29], so one explanation for differences in the diagnostic intervals may be differences in the cancer stage distribution. Staging information availability varied across provinces, preventing our ability to look at this factor. We expect that, at the population level, stage distribution differences would be small, particularly since we stratified our analyses by detection method.

## 5. Conclusions

In conclusion, our results highlight clinically important differences in the length of the breast cancer diagnostic interval across and within provinces in Canada and by detection method. The length of the diagnostic interval was associated with increasing comorbid disease burden and patients living in rural areas generally had longer diagnostic intervals. Variability across provinces in the presence of and/or size of these associations underscores the context-specific nature of diagnostic systems and processes. Improved geographic distribution of services and increased awareness of the obfuscating role of comorbid disease on cancer detection are two areas for improvement identified by this work. Routine surveillance of the diagnostic interval should be implemented by provincial cancer agencies charged with ensuring high quality cancer care. Attention should be paid to developing more personalized, tailored services so that equitable access to a timely diagnosis can be achieved.

## Figures and Tables

**Table 1:** Screen-Detected Group: population characteristics by province (%).

Variables	British Columbia	Alberta	Manitoba	Ontario	Nova Scotia
	n = 4947	n = 3780	n = 1067	n = 12,962	n = 1525
**Age**					
<40	0	0	0	0.6	0
40–49	14	15.9	0.6	4	15.9
50–59	28.2	29.9	38.9	33.1	26.2
60–69	33.4	30.4	48.9	39.7	37.3
70–74	12.7	10.9	9.9	13.8	10.6
75+	11.7	12.9	1.7	8.8	10
**Co-morbidities**					
0–3 ADGs	28.9	-	21	23.9	16.8
4–5 ADGs	25.3		24.7	24.4	19.7
6–7 ADGs	22.1		21.7	22	22.9
8–9 ADGs	13.1		16.1	15.4	19.9
10+ ADGs	10.6		16.4	14.3	20.7
**SES-Rurality**					
High/Urban	20.4	20.1	15.1	20.7	15.5
Med/Urban	53.5	48	43	52	40.1
Low/Urban	13.5	13	9.1	13.2	9.4
High/Rural	2.6	3.6	8	2.6	7.3
Med/Rural	7.3	11.2	20.7	8.3	21
Low/Rural	2.7	3.7	4.1	2.9	6.5
Missing/Unknown	0	0.3	0	0.3	0
**UPC index score**					
0 visits	5.7	3.1	4.9	5.8	2.3
1–2 visits	7.6	7.6	7.4	10.4	10.3
UPC ≤ 0.75 **(Low)**	40.2	44.9	36.5	25.1	25.1
UPC > 0.75 **(High)**	46.4	44.4	51.3	58.7	62.4

ADGs = Aggregate Diagnosis Groups; SES = Socioeconomic status; UPC = Usual Provider of Care Index.

**Table 2:** Non-Screen-Detected Group: population characteristics by province (%).

Variables	British Columbia	Alberta	Manitoba	Ontario	Nova Scotia
	n = 9251	n = 7005	n = 3017	n = 31,475	n = 2277
**Age**					
<40	6.2	8.5	6.2	6.7	6.3
40–49	18	23.8	20.5	22.9	16.6
50–59	21.7	24	19.2	22.2	19.5
60–69	21.5	17.6	17.7	19.5	20.1
70–74	8	7.3	9	7.6	9.6
75+	24.5	18.8	27.4	21.2	27.9
**Co-morbidities**					
0–3 ADGs	33.8	-	24.6	26.3	25.3
4–5 ADGs	23.7		21.8	22.5	21.7
6–7 ADGs	18.4		20.3	21.1	18.4
8–9 ADGs	12.8		16.2	15	14.6
10+ ADGs	11.3		17.1	15.1	19.9
**SES-Rurality**					
High/Urban	17.6	16.4	14.8	19.6	11.9
Med/Urban	50.8	45.8	45	52.9	39.7
Low/Urban	18	14.3	12.9	15.6	12.2
High/Rural	2.7	4.1	5.5	2.2	5.8
Med/Rural	8.2	14.2	17.3	7.1	22.6
Low/Rural	2.6	4.6	4.3	2.2	7.6
Missing/Unknown	0	0.5	0	0.3	0
**UPC index score**					
0 visits	7.4	6.5	6.6	7.2	7.2
1–2 visits	8.3	8.4	9.2	10.8	13.7
UPC ≤ 0.75 **(Low)**	40	46.8	38.2	27.6	25.3
UPC > 0.75 **(High)**	44.4	38.4	45.9	54.4	53.8

ADGs = Aggregate Diagnosis Groups; SES = Socioeconomic status; UPC = Usual Provider of Care Index.

**Table 3:** Diagnostic interval length median, interquartile range and 90th percentile for screen- and non-screen-detected cancers across the 5 provinces (days).

	British Columbia	Alberta	Manitoba	Ontario	Nova Scotia
	n = 14,198	n = 12,373	n = 4216	n = 44,437	n = 3802
**Screen-detected**					
Median	30	19	30	28	36
IQR	17–55	NA	21–47	16–48	23–56
90th %	81	70	71	75	84
**Non-screen-detected**					
Median	32	21	37	34	24
IQR	14–64	NA	21–68	16–68	13–47
90th %	123	92	112	126	85

IQR = Interquartile range.

**Table 4:** Screen-Detected Group: Diagnostic interval differences by province at the median and 90th percentiles (days) *.

	British Columbia		Alberta		Manitoba		Ontario		Nova Scotia	
Variables	Median	90th	Median	90th	Median	90th	Median	90th	Median	90th
Intercept	25	59	13	75	26.4	69	24	60	NA	NA
**Age**										
<40	-	-	-	-	-	-	**−11.3**	−18	-	-
40–49	3.0	7.2	2	22	8.8	−1	**−5**	**28**	0.1	15.8
50–59	1	0	1	1	2.8	2.5	0	3	0.7	−1.9
60–69	Ref	Ref	Ref	Ref	Ref	Ref	Ref	Ref	Ref	Ref
70–74	3	−0.2	1	0.2	−1.6	−10	0.4	4	−0.1	−5.6
75+	0	−4.2	1	−3.3	4.5	13	0.6	−2	3.3	0.8
**Co–morbidities**										
0–3 ADGs	Ref	Ref	- **	- **	Ref	Ref	Ref	Ref	Ref	Ref
4–5 ADGs	1	1			−1.2	−15	−1	−3	−1.1	5
6–7 ADGs	0	3			−0.8	−11.5	0	−3	−2.4	7.4
8–9 ADGs	2	7.6			1.8	−5.5	1.4	1	0.4	14.6
10+ ADGs	3	11.2			−0.3	−6	0	3	0.9	4.8
**SES–Rurality**										
High/Urban	Ref	Ref	Ref	Ref	Ref	Ref	Ref	Ref	Ref	Ref
Med/Urban	−1	5.2	2	−10.3	3.1	3	1.4	5	−0.9	−8.4
Low/Urban	1	**16.6**	2	−12.3	−0.5	−4.5	1.4	3	−5	13.4
High/Rural	0	12.4	8	−10.3	1.6	−2.5	3.3	**15**	10.4	0.3
Med/Rural	6	6.2	**12**	−2.2	1.5	12.5	**4**	**12**	**11.9**	13.1
Low/Rural	10.2	0.8	**11**	−8.7	5.3	14	5.4	17	**19.3**	26.6
**UPC index score**										
0 visits	−5	−6.2	−3	−1.8	−3.5	−4	−1.4	−2	−8.6	8
1–2 visits	−1	−1	−1	−3.8	−2.5	−5	**−2.6**	−8.6	−3.3	1.1
UPC < 0.75	−1	−0.4	0	5.2	−1.5	−3.5	−1.4	−1	−1.3	15
UPC > 0.75	Ref	Ref	Ref	Ref	Ref	Ref	Ref	Ref	Ref	Ref

* Results underlined had 0.001 < *p*-value < 0.05. Bold font results had a *p*-value of <0.001. ** Alberta results controlled for Charlson Comorbidity index (results not reported) rather than ADGs. ADGs = Aggregate Diagnosis Groups; Ref = Reference category; SES = Socioeconomic status; UPC = Usual Provider of Care Index.

**Table 5:** Non-Screen Detected Group: Diagnostic interval differences by province at the median and 90th percentiles (days) *.

	British Columbia		Alberta		Manitoba		Ontario		Nova Scotia	
Variables	Median	90th	Median	90th	Median	90th	Median	90th	Median	90th
Intercept	25.6	98	19	72	35	102	28	104.7	NA	NA
**Age**										
<40			0	−3	1	−2.8	**−4**	−5	0.4	−10.8
40–49	2.3	2	−0.3	9	3.5	0.8	0	5	0.5	4
50–59	0.5	2.2	0.4	13	0.5	−2.5	0	7.3	−0.1	−3
60–69	Ref	Ref	Ref	Ref	Ref	Ref	Ref	Ref	Ref	Ref
70–74	−1.3	−6.6	1.7	13	−1.5	−20.8	−1	−12.7	−2.2	6
75+	−2	−7.7	−1	−12	−3.5	−19	**−6**	**−30**	−2.4	−12.8
**Co–morbidities**										
0–3 ADGs	Ref	Ref	- **	- **	Ref	Ref	Ref	Ref	Ref	Ref
4–5 ADGs	3.8	16.9			**6.5**	16.3	**3**	**12**	−0.1	4.8
6–7 ADGs	**5.4**	**25.9**			5.5	**37.3**	**4**	**18**	0.7	10.8
8–9 ADGs	**6.3**	**25.2**			7.5	15.5	**5**	**13.3**	4.4	11.8
10+ ADGs	**7.9**	**36.7**			**8.5**	15.5	**7**	**23**	4.5	24.8
**SES–Rurality**										
High/Urban	Ref	Ref	Ref	Ref	Ref	Ref	Ref	Ref	Ref	Ref
Med/Urban	1.2	3.8	0.2	−5	−1	−9.8	**2**	−3	−2.2	−8.3
Low/Urban	0.3	−4.2	−0.2	−13	1	−3.8	2	−7.3	−2.7	−21
High/Rural	−6.6	4.7	−4.4	−14	−4.5	−11.5	3	5	1.1	−14
Med/Rural	−2.8	−2.2	−0.1	−11	−5.5	−6	0	−3.3	3.3	−13.8
Low/Rural	−1.9	−20.9	−0.2	−7	−3	5.5	−1	−8	5.9	2
**UPC index score**										
0 visits	−4.3	−6.3	**−4.3**	**−25**	−3.5	−7.8	**−7**	**−27.3**	−5.8	0.8
1–2 visits	−2.3	−6.8	−0.4	−11	2.5	0	**−3**	−2	−5	9.3
UPC < 0.75	−0.5	3.5	0.8	6	−0.5	5	0	6.3	0.5	3.3
UPC > 0.75	Ref	Ref	Ref	Ref	Ref	Ref	Ref	Ref	Ref	Ref

* Results underlined had 0.001 < *p*-value < 0.05. Bold font results had a *p*-value of <0.001. ** Alberta results controlled for Charlson Comorbidity index (results not reported) rather than ADGs. ADGs = Aggregate Diagnosis Groups; Ref = Reference category; SES = Socioeconomic status; UPC = Usual Provider of Care Index.

**Figure A1:**
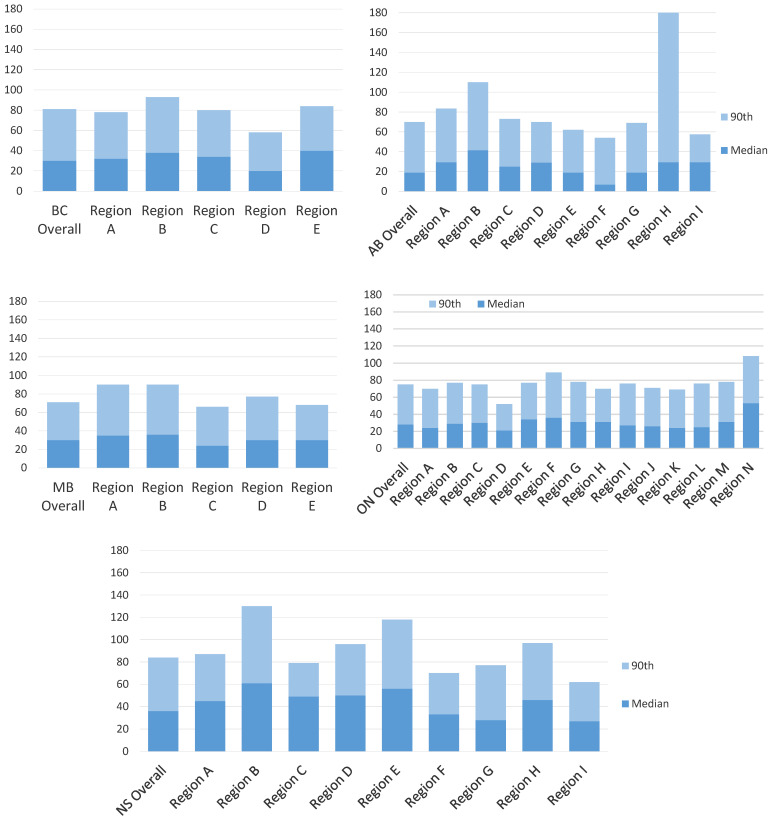
Intra-provincial variation in diagnostic interval length median and 90th percentile range for screen-detected cancers. AB = Alberta; BC = British Columbia; MB = Manitoba; ON = Ontario; NS = Nova Scotia.

**Figure A2:**
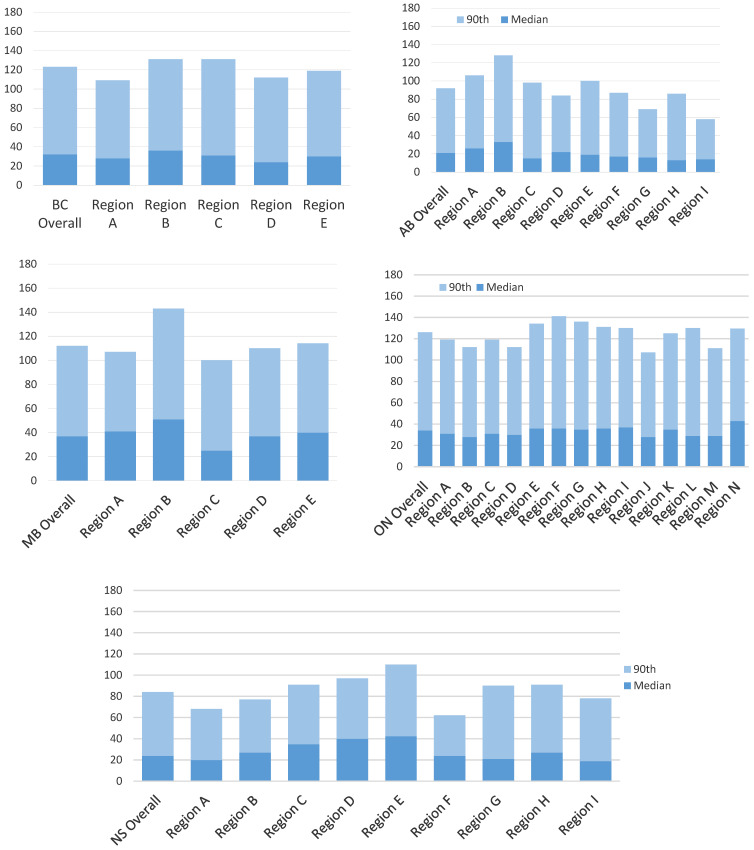
Intra-provincial variation in diagnostic interval length median and 90th percentile Range for non-screen detected cancers. AB = Alberta; BC = British Columbia; MB = Manitoba; ON = Ontario; NS = Nova Scotia.

## Data Availability

The data supporting the conclusions of this article are not available in a public repository, in accordance with provincial government policies. They are housed at Population Data BC (British Columbia), Manitoba Population Research Data Repository (Manitoba), Institute for Clinical Evaluative Sciences (Ontario), and Health Data Nova Scotia (Nova Scotia). Nova Scotia data were provided by Health Data Nova Scotia and the Nova Scotia Department of Health and Wellness (Cancer Care NS and NS Breast Screening Program), however, the observations and opinions expressed are those of the authors and do not represent those of either Health Data Nova Scotia or the Nova Scotia Department of Health and Wellness. Data for this study were also provided by Population Data BC and BC Cancer. All inferences, opinions, and conclusions drawn in this study are those of the authors, and do not reflect the opinions or policies of the BC Data Steward(s). (“BC Cancer Agency Registry Data. V2, Population Data BC: BC Cancer Agency; 2011 [Available from: https://www.popdata.bc.ca/data, (accessed on 4 March 2022)],” n.d.; “Medical Services Plan (MSP) Payment Information File. V2, MOH (2011): British Columbia Ministry of Health; 2011 [Available from: https://www.popdata.bc.ca/data, (accessed on 4 March 2022)],” n.d.; “Consolidation File (MSP Registration & Premium Billing). V2, Population Data BC: British Columbia Ministry of Health (2011); 2011 [Available from: https://www.popdata.bc.ca/data, (accessed on 4 March 2022)],” n.d.). The Ontario data repository for this study was supported by ICES, which is funded by an annual grant from the Ontario Ministry of Health (MOH) and the Ministry of Long-Term Care (MLTC). Parts of this material are based on data and information compiled and provided by Ontario Health (OH) and the Canadian Institute for Health Information (CIHI). The analyses, conclusions, opinions and statements expressed herein are solely those of the authors and do not necessarily reflect those of the data sources; no endorsement is intended or should be inferred. The authors gratefully acknowledge CancerCare Manitoba for their ongoing support and Manitoba Health for the provision of data. The results and conclusions presented are those of the authors. No official endorsement by Manitoba Health is intended or should be inferred.

## Appendix A. Relevant Healthcare Administrative Data Codes

Codes used to identify first ever* breast cancer diagnosis:

- Histologically confirmed invasive breast cancer (behaviour = 3, ICD-0; 174.0–174.9 ICD-9)
- * For patients with more than one cancer diagnoses in the same breast on the same day, select based on the order of the below criteria:
  Pick case with the highest stage;
  Pick case with highest histology priority;
  Pick case with the first malignancy number.

Codes used to identify diagnostic tests and breast-cancer related visits (for the small subset of patients with no diagnostic tests):

- Breast cancer: OHIP: 174, 175 CIHI**: C50^
- Other related cancer: OHIP: 162, 170, 173 195, 196, 197, 198, 199, CIHI: C34.90 C44.5 C76.1 C76.4 C77.3 C78.0 C78.2 C78.7 C79.2 C79.3^ C79.5^ C79.8^ C79.9 C80^
- Benign breast neoplasm/CIS: OHIP: 214, 217, 229, 232, 233, 234, 238, 239 CIHI: D17.1 D24^ D04.5 D05^ D48.6^
- Infectious/inflammatory conditions of the breast: OHIP: 610, 611 CIHI:N61
- Breast biopsy (with/without ultrasound guidance): OHIP: J149, R107, X121, Z141, Z143, E525, E542 CCI: 2YK71, 2YM71, 2MD71, 3YM12 3YM94
- Cyst aspiration or drainage: OHIP: Z118, Z139, Z140
- Mastectomy—any type: OHIP: R105, R108, R109, R111, R117 CCI: 1YK87 1YL87 1YL89 1YM87 1YM89 1YM90 1YM91
- Surgical consult with no procedure: OHIP: A035, A935
- Bilateral mammography: X185 CCI: 3YM10
- Diagnostic mammography and related procedures: X184, J004, J037, X192, X194, X201 CCI: 3YL10
- Opportunistic screening mammogram: OHIP: X172↑↑, X178↑↑
- Breast ultrasound: OHIP: J127, J427 CCI: 3YM30
- Breast MRI: OHIP: X446, X447 and for 2007: X441, X445 CCI: 3YM40
- Other ultrasound: J182, J195, J202, J425, J482, J502, CCI: 3GY30
- Other MRI: X421, X425, (Post 2007: X441, X445), X471, X475, X490, X492, X499 CCI: 3AN40
- Nuclear medicine: J650, J666, J667, J850 CCI: 3YM70
- Abnormal mammogram within breast screening program: Screened = 2 (mammogram only) or 3 (yes, both PE and mammogram) and Finalres = C (breast cancer)
- How codes were used:
- Collected all breast-related diagnostic encounters listed above in 6 months from the diagnosis date (including the date of diagnosis).

(1) For screening tests: In 6 months before (and including) the date of diagnosis, identify the earliest screening mammogram, including:
  - Abnormal mammograms within the breast screening program; and
  - Opportunistic screening mammograms
(2) For diagnostic tests: In 6 months before (and including) the date of diagnosis, use OHIP and CIHI-DAD data, identify the earliest date of the last visits to referring physician who ordered the first test of each procedure below:
  - Diagnostic mammograms
  - Non-specific mammograms
  - Breast ultrasound
  - Breast MRI
  - Breast biopsy
  - Breast surgeon consultation PLUS codes for breast cancer, benign neoplasms/CIS or infectious/inflammatory conditions
(3) For breast-related ED visits:
  - Breast cancer
  - Benign neoplasms/CIS
  - Infectious/inflammatory conditions, breast

OTo calculate diagnostic interval (in days):

Defined as the time interval between the index contact date and the diagnosis date.

The index contact date was determined using algorithms specific to the detection method. For screen-detected patients, the index contact date is the date of initial screening. For non-screened patients, the index contact date is the earliest date of the following:

- Most recent healthcare encounter with referring physician prior to the first diagnostic test
- The earliest date of breast-related encounters in 6 months before (including) the date of diagnosis

**Table A1:** Translation of diagnosis codes to ICD-10 equivalent.

OHIP Code	Disease Diagnosis	ICD 10 Equivalent Code *
162	Lung neoplasm	C34.90
170	Bone neoplasm	N/A **
173	Other skin malignancies	C44.5
174	Female breast neoplasm	C50 ^
175	Male breast neoplasm	C50 ^
195	Malignant neoplasms—Other ill defined sites	C76.1 C76.4
196	Secondary neoplasms of lymph nodes	C77.3
197	Secondary neoplasm of respiratory and digestive	C78.0 C78.2 C78.7
198	Malignant neoplasms—metastatic or secondary, carcinoma	C79.2 C79.3 ^ C79.5 ^ C79.8 ^ C79.9
199	Other malignant neoplasms	C80 ^
214	Malignant neoplasms—lipoma	D17.1
217	Benign neoplasms—breast	D24 ^
228	[Haemangioma] and lymphangioma	N/A
229	Other benign neoplasms	N/A
232	CIS—Skin	D04.5
233	CIS—Breast and [genito-urinary system]	D05^
234	CIS—Other	N/A
238	Neoplasms uncertain behavior—other & unspecified sites	D48.6 ^
239	Unspecified neoplasms eg polycythemia vera	N/A
457	Lymphedema, lymphangitis	N/A
610	Cystic mastitis, fibroadenosis of breast	N/A
611	Breast abscess, gynecomastia, hypertrophy, other breast	N/A
683	Acute lymphadenitis	L04.2
A035	General surgery consultation	
A935	General surgery special surgery consultation	
E525	Breast excision: Tumour or tissue for diagnostic biopsy and/or treatment, e.g.,carcinoma, fibroadenoma or fibrocystic disease after mammographic localization, add $ to R107	
E542	Needle biopsy when performed outside hospital, add $ to Z141	
J004	Embolization of spinal arteriovenous malformation: intramammary needling for localization under mammographic control	
J037	Lymphangiogram: mammary ductography	
J127	Diag US: scan B-mode (per breast)	3YM30
J149	Ultrasonic guidance of biopsy, aspiration, amniocentesis or drainage procedures (one physician only)	
J182	Diag US Extremities: per limb (excluding vascular study)	
J195	Diag US Vascular: peri-art anal freq anal + scan—per limb Not in April 2013 OHIP Schedule	
J202	Diag US Vascular: duplex scan i.e., simultaneous real time, B-mode imaging and frequency/spectral analysis, unilateral	
J425	Diag US Thorax etc: Chest masses, pleural effusion—A & B-mode	3GY30
J427	Diag US: scan B-mode (per breast)	3ym30 needs to be here and look for other synonyms for the other CIHI codes
J482	Diag US Extremities: per limb (excluding vascular study)	
J502	Diag US Vascular: duplex scan i.e., simultaneous real time, B-mode imaging and frequency/spectral analysis, unilateral	
J650	Nuclear Muskuloskeletal: bone scintigraphy general survey	
J666	Nuclear Tomography: maximum one per Nuclear Medicine examination	3YM70
J667	Nuclear Cardiovascular: first transit with blood pool images	
J850	Nuclear Muskuloskeletal: bone scintigraphy general survey	
R105	Breast excision: partial mastectomy plus radical node dissection Not in April 2013 OHIP Schedule	
R107	Breast excision: Tumour or tissue for diagnostic biopsy and/or treatment, e.g.,carcinoma, fibroadenoma or fibrocystic disease	
R108	Breast mastectomy—female w/wo biopsy—simple	1YM89 1YM90
R109	Breast mastectomy—female w/wo biopsy—radical or modified radical	1YM91
R111	Breast excision: partial mastectomy or wedge resection for treatment of breast disease, with or without biopsy, e.g., carcinoma or extensive fibrocystic disease	1YK87 1YL87 1YL89 1YM87
R117	Breast mastectomy—female w/wo biopsy—subcutaneous with nipple preservation	
X121	Xray special examinations: bronchogram stereotactic core breast biopsy	3YM12 3YM94
X172 *	Mammogram—no signs or symptoms—dedicated equipment—unilateral	
X178 *	Mammogram—no signs or symptoms—dedicated equipment—bilateral	
X184 **	Mammogram—signs or symptoms—unilateral	
X185 **	Mammogram—signs or symptoms—bilateral	3YM10
X192	Xray: Misc exams—mammary ductography	3YL10
X194 *	Mammogram—no signs or symptoms—additional cone view w/wo magnification (limit two per breast)	
X201	Mammogram—no signs or symptoms—breast biopsy specimen X-ray	
X421	MRI head multislice sequence	3AN40
X425	MRI head repeat	
X441	MRI thorax multislice sequence	
X445	MRI thorax repeat	
X446	MRI breast—unilateral or bilateral—multislice sequence	3YM40
X447	MRI breast—repeat	
X471	MRI extremity or joint—multislice sequence	
X475	MRI extremity or joint—repeat	
X490	MRI limited spine—multislice sequence	
X492	MRI limited spine—repeat	
X499	MRI complex spine—3D MRI acquisition sequence	
Z118	Skin/subcutaneous operation: foreign body removal—aspiration of superficial lump for cytology	
Z139	Operations of the breast: aspiration of cyst—one or more	
Z140	Operations of the breast: drainage of intramammary abscess or haematoma—single or multilocated—local anaesthetic	
Z141	Operations of the breast: needle biopsy—one or more	2YK71 2YM71 2MD71
Z143	Operations of the breast: needle biopsy—large core biopsy	

* Used ICD9 (basis for OHIP codes) converter to ICD-10 on ICD10Data.com In some instances only the more breast/cancer specific subcodes were included. ICD-9 781, 785 and 788 that mention ‘masses’ have no cancer-related subcodes in ICD-10. ** N/A means there were no observations of ICD-10 equivalent codes in the CIHI DAD and NACRS data for our cohort. ^ is used to indicate that all subcodes should be included.

## Appendix D. Inter- and Intra-Provincial Variation in the Diagnostic Interval

**Table A2:** Inter- and intra-provincial variation in the diagnostic Interval median and 90th percentile by detection method.

	Screen-Detected Range (Days)		Non-Screen Detected Range (Days)	
	Median Range	90th Range	Median Range	90th Range
Inter-provincial	17	14	13	42
British Columbia Intra-provincial	20	35	22	22
Alberta Intra-provincial	34	127	20	30
Manitoba Intra-provincial	22	24	26	43
Ontario Intra-provincial	29	56	15	34
Nova Scotia Intra-provincial	29	56	24	48
